# Identification of a Tsal1_52–75_ salivary synthetic peptide to monitor cattle exposure to tsetse flies

**DOI:** 10.1186/s13071-016-1414-8

**Published:** 2016-03-15

**Authors:** Martin Bienvenu Somda, Sylvie Cornelie, Zakaria Bengaly, Françoise Mathieu-Daudé, Anne Poinsignon, Emilie Dama, Jeremy Bouyer, Issa Sidibé, Edith Demettre, Martial Seveno, Franck Remoué, Antoine Sanon, Bruno Bucheton

**Affiliations:** Centre International de Recherche-Développement sur l’Elevage en zone Subhumide (CIRDES), 01 BP 454 Bobo-Dioulasso 01, Burkina Faso; Université Polytechnique de Bobo-Dioulasso, 01 BP 1 091 Bobo-Dioulasso 01, Burkina Faso; Institut de Recherche pour le Développement (IRD), Unité Mixte de Recherche 224, Maladies Infectieuses et Vecteurs: Ecologie, Génétique, Evolution et Contrôle (MIVEGEC), Montpellier, 34394 Cedex 5 France; CIRAD, UMR CIRAD-INRA Contrôle des Maladies Animales, Campus International de Baillarguet, F34398 Montpellier, France; Pan African Tsetse and Trypanosomosis Eradication Campaign (PATTEC), Projet de Création de Zones Libérées Durablement de Tsé-tsé et de Trypanosomoses (PCZLD), Bobo-Dioulasso, Burkina Faso; Institut de Génomique Fonctionnelle, CNRS UMR 5203, INSERM U1191, UM1, UM2, Plate-forme de Protéomique Fonctionnelle CNRS UMS BioCampus 3426, 34094 Montpellier, France; Université de Ouagadougou, UFR/SVT, Laboratoire d’Entomologie Fondamentale et Appliquée (LEFA), BP 9499 Ouagadougou 06, Burkina Faso; Institut de Recherche pour le Développement, Unité Mixte de Recherche IRD-CIRAD 177, Interactions hôtes-vecteurs-parasites dans les maladies dues aux Trypanosomatidae, Campus International de Baillarguet, Montpellier, 34398 Cedex 5 France

**Keywords:** Synthetic Peptide, Biomarker of Exposure, Cattle, Tsetse Flies, African Animal Trypanosomosis

## Abstract

**Background:**

The saliva of tsetse flies contains a cocktail of bioactive molecules inducing specific antibody responses in hosts exposed to bites. We have previously shown that an indirect-ELISA test using whole salivary extracts from *Glossina morsitans submorsitans* was able to discriminate between (i) cattle from tsetse infested and tsetse free areas and (ii) animals experimentally exposed to low or high numbers of tsetse flies. In the present study, our aim was to identify specific salivary synthetic peptides that could be used to develop simple immunoassays to measure cattle exposure to tsetse flies.

**Methods:**

In a first step, 2D-electrophoresis immunoblotting, using sera from animals exposed to a variety of bloodsucking arthropods, was performed to identify specific salivary proteins recognised in cattle exposed to tsetse bites. Linear epitope prediction software and Blast analysis were then used to design synthetic peptides within the identified salivary proteins. Finally, candidate peptides were tested by indirect-ELISA on serum samples from tsetse infested and tsetse free areas, and from exposure experiments.

**Results:**

The combined immunoblotting and bioinformatics analyses led to the identification of five peptides carrying putative linear epitopes within two salivary proteins: the tsetse salivary gland protein 1 (Tsal1) and the Salivary Secreted Adenosine (SSA). Of these, two were synthesised and tested further based on the absence of sequence homology with other arthropods or pathogen species. IgG responses to the Tsal1_52–75_ synthetic peptide were shown to be specific of tsetse exposure in both naturally and experimentally exposed hosts. Nevertheless, anti-Tsal1_52–75_ IgG responses were absent in animals exposed to high tsetse biting rates.

**Conclusions:**

These results suggest that Tsal1_52–75_ specific antibodies represent a biomarker of low cattle exposure to tsetse fly. These results are discussed in the light of the other available tsetse saliva based-immunoassays and in the perspective of developing a simple serological tool for tsetse eradication campaigns to assess the tsetse free status or to detect tsetse reemergence in previously cleared areas.

**Electronic supplementary material:**

The online version of this article (doi:10.1186/s13071-016-1414-8) contains supplementary material, which is available to authorized users.

## Background

African trypanosomosis, a parasitic vector-borne disease that constitutes a major constraint to development in sub-Saharan Africa, exists under two forms: Human African Trypanosomosis (HAT) known also as sleeping sickness and African Animal Trypanosomosis (AAT) or *Nagana.* Tsetse flies (*Glossina* spp.) are the cyclical vectors of the trypanosome species causing these diseases. Some 46 million cattle distributed over 10 million km^2^ in 38 sub-Saharan African countries [1, 2] are estimated to be at risk of contracting AAT and hamper significantly the socio-economic development of these African regions [3, 4]. Among the 38 tsetse-infested countries, 34 are amongst the poorest in the world [5] and have included tsetse and trypanosomosis as a constraint in their poverty reduction strategy papers under the heavily indebted poor countries initiative [6].

The main strategies used to control or eradicate AAT remain (i) chemoprophylaxis and chemotherapy with trypanocidal drugs, (ii) promoting trypanotolerant cattle, and (iii) tsetse control or eradication programmes [1]. The Pan African Tsetse and Trypanosomiasis Eradication Campaign (PATTEC) initiative promoting integrated control of AAT and large tsetse eradication campaigns are underway in Uganda, Kenya and Ethiopia in East Africa and in Ghana, Burkina Faso and Mali in West Africa, with the aim of improving breeding and agriculture by creating new tsetse free areas [7].

Entomological evaluation tools represent an important component of any vector based control programme to appropriately target implementation areas and to evaluate their efficacy in time [8]. To date, the conventional method used within tsetse eradication campaigns is to estimate tsetse fly densities with traps deployed at fixed or temporary sites [9]. Important constraints are nevertheless associated with this method. The deployment and monitoring of traps, most of the time in very large areas (20 km/day/person in walking) with poor accessibility, is costly and demanding in terms of human resources and logistics. It is also known that traps have a poor efficiency (below 1 % of the flies present in a 1 km^2^ around the trap are captured daily) and are becoming even less efficient at low tsetse densities [8, 10, 11]. Furthermore, traps are generally set up in sentinel fixed sites and thus only provide an indirect estimate of cattle exposure to tsetse bites especially in agro-pastoral areas where herds are very mobile. Alternative methods based on the evaluation of the antibody (Ab) responses directed against bloodsucking arthropod salivary antigens have been developed in the last few years. During the blood meal, hematophagous arthropods inject a mixture of anti-haemostatic, anti-inflammatory and immunomodulatory molecules into the skin of their hosts. These molecules play a crucial role in achieving an effective blood meal [12], but also in the establishment or not of pathogens into the vertebrate host [13]. The antigenic properties of these molecules have also been used to develop a range of immunoassays to detect associated specific Abs and to assess host exposure to a range of arthropod vectors of human pathogens [14].

Recent studies have shown that the human IgG response against whole salivary extracts (WSE) of several *Glossina* (*G.*) species (*G. morsitans* (*m.*) *morsitans*, *G. fuscipes* (*f.*) *fuscipes* and *G. palpalis* (*p.*) *gambiensis*) was correlated with human exposure to tsetse flies in different HAT endemic areas [15–18]. Similar results were observed in outbred cattle from a tsetse infested area in South-West Burkina Faso that were shown to harbor higher IgG responses than cattle in the North where tsetse flies are absent. These results were further confirmed in cows experimentally exposed to the bite of a range of *Glossina* species and other bloodsucking arthropods [19]. However, the use of WSE in immunoassays is likely impaired by the existence of potential cross-reactions with Abs directed against common saliva antigens that are shared by different arthropod species [20]. Other important drawbacks are the difficulty in achieving mass production of WSE in a standardized manner and the storage of these antigens over long periods. WSE are thus poorly suited for large scale studies [21, 22]. Resorting to recombinant salivary proteins has enabled to overcome some of these limitations and a number of immunoassays based on specific recombinant salivary proteins have been developed to detect exposure to a range of arthropods [23–27]. An alternative strategy has been to identify specific linear epitopes by *in silico* approaches in order to design peptides to be used as the immunoassay antigens. Production of short synthetic peptides at high purity can easily be entrusted to a private company; shipment and storage are facilitated as they can be lyophilised. In the last few years, such approaches were successfully applied to develop salivary biomarkers of human exposure to *Aedes aegypti* [28] and *Anopheles* (*An.*)*. gambiae* [21, 22]. Recently, the IgG response to a synthetic peptide designed from the Tsetse Saliva Growth Factor-1 (Tsgf1) sequence, was shown to be specific of human exposure to tsetse flies [29] and was successfully used to assess the evolution of human tsetse contacts during a vector control intervention in Guinea [30].

In the present study, our aim was to design peptides that could be used to assess exposure of cattle to tsetse flies. An immuno-proteomic approach, using sera from animals experimentally exposed to several arthropod species and WSE from *G. m. submorsitans*, was first used to identify the most specific immunogenic salivary proteins to detect *Glossina* exposure. Several epitope prediction and protein conformation software were then used in combination with Blast analysis to design linear peptides within the identified proteins. Synthetic peptides were finally produced and evaluated in an indirect-ELISA test with serum samples from (i) cattle from tsetse free or tsetse infested areas and (ii) cows experimentally exposed to low or high numbers of tsetse flies.

## Methods

### Serum samples

All bovine serum samples used in this study were collected from cattle outbred in different environmental settings from Burkina Faso or were obtained by experimental exposure of cows to several arthropod species and are described in detail elsewhere [19]. Samples from outbred cattle included 17 samples collected in a tsetse free area in Northern Burkina Faso and 43 samples collected in a tsetse infested area in the South-West part of the country. Concerning experimentally exposed sera, we included six samples collected from six cows individually exposed weekly to the bite of *G. m. submorsitans*, *G. p. gambiensis*, *Amblyomma* (*A.*) *variegatum*, *An. gambiae*, *Tabanidae* spp. or *Stomoxys* spp. Samples used in the present study were those collected at the end of the exposure experiments, after 12 weeks for *An. gambiae* and 23 weeks for all other species. In addition we also used 96 serum samples collected weekly from two groups of four animals that were exposed to 50 *G. m. submorsitans* flies twice a week during 11 weeks (high exposure group) or 10 flies weekly during the same period (low exposure group).

### Production of whole salivary extracts

WSE were obtained from 10–12 day-old *G. m. submorsitans* uninfected males and females from the IRD/CIRAD colony in Montpellier (France). The tsetse saliva was collected as described previously [17] by a salivation technique that does not require the dissection of salivary glands to avoid the presence of non-salivary antigens in WSE. Briefly, tsetse flies (4–6 flies) were enclosed in 50 ml Falcon tubes closed by a mosquito net and placed above a drop of salivation buffer on warm slides. Buffer drops were collected after 10 min of salivation and were stored at −80 °C before use. Prior to electrophoresis, WSE were desalted and concentrated using the 2-D Clean-Up Kit (GE Healthcare, Germany) according the manufacturer instructions. Protein concentrations of WSE were assessed by the Bradford method.

### Identification of salivary proteins

#### Two-dimensional electrophoresis (2DE) and gel staining

For the first dimension electrophoresis, isoelectric focusing (IEF) was carried out with 60 μg of *G. m. submorsitans* WSE on 11 cm pH 3–11 non linear immobiline^TM^ dryStrips (GE Healthcare, Germany). Strips were rehydrated for 10–20 h at room temperature with protein sample made up to 170 μl in IEF buffer (7 M urea, 2 M thiourea, 4 % CHAPS, 0.2 % tergitol, 0.8 % IPG buffer, 1 % octylβglucoside and 2 % DeStreak reagent). The IEF conditions were: temperature 20 °C; current 50 μA per strip; 60 V (step’n’hold) for 1 h; 500 V (gradient) for 1 h; 1 000 V (gradient) for 1 h; 6 000 V (gradient) for 2 h and then 6 000 V holds to 30 000 Vhs. After the IEF, the strips were reduced for 10 min with 65 mM DTT buffer pH 6.8 (50 mM Tris HCl pH 6.8, 6 M urea, 30 % Glycerol, 2 % SDS) and alkylated for 15 min with 81 mM of iodoacetamide buffer pH 8.8 (50 mM Tris HCl pH 8.8, 6 M urea, 30 % Glycerol, 2 % SDS). Then equilibrated strips in pH 6.8 buffer (50 mM Tris HCl pH 6.8, 6 M urea, 30 % Glycerol, 2 % SDS) were applied to 10-20 % SDS-PAGE Tris–HCl gels (Criterion, Biorad) for second dimension and sealed with agarose (1 % agarose low melting, 0.2 % SDS and 150 mM Tris pH 6.8). 2DE-gels were run at 30 V for 20 min and then 200 V for 55 min.

After the second dimension, a preparative 2DE-gel was fixed for 20 min in 50 % ethanol/5 % acetic acid solution, and then 10 min in 50 % ethanol solution, washed three times in ultrapure water. Finally, this gel was stained with a colloidal blue solution (Fermentas, Saint-Remy les Chevreuse, France) overnight and washed four times in ultrapure water.

#### Identification of *G. m. submorsitans* salivary proteins by mass spectrometry

Visible spots after colloidal blue staining were manually excised under a laminar flow hood and enzymatic in-gel digestion was performed automatically (Tecan freedom evo® proteomics) according to the Shevchenko modified protocol [31].

Briefly, protein spots were digested using 150 ng of trypsin, peptide extraction was performed using 5 sonication cycles of 2 min each and peptides were concentrated 1 h at 50 °C in a heat block. Peptide samples were automatically spotted (Tecan freedom evo® proteomics). For this step, 0.5 μl of peptide sample and 0.5 μl of α-cyano-4-hydroxy-trans-cinnamic acid (a saturated solution prepared in acetonitrile/trifluoroacetic acid, 50: 0.1 %, vortexed, sonicated 30 s and microcentrifuged 30 s with a 1/3 dilution of the supernatant used as the matrix) were deposited on a 384-well MALDI anchorship target using the dry-droplet procedure [32] and air dried at room temperature. Peptide samples were then desalted using a 10 mM phosphate buffer and dried again at room temperature. MALDI-TOF MS analysis was performed using UltraFlex MALDI TOF-TOF mass spectrometer (Brucker Daltonics, Bremen, Germany) in the reflecton mode with a 26 kV accelerating voltage and a 50 ns delayed extraction. The AutoXecute™ module of Flexcontrol™ v3.0 (Bruker Daltonics) (laser power ranged from 40 to 50 %, 600 shots) was used to acquire mass spectra. Spectra were analysed using FlexAnalysis™ software v3.0 (Bruker Daltonics) and calibrated internally with the autoproteolysis peptides of trypsin (m/z: 842.51; 1045.556; 2211.10). Peptides were selected in the mass range of 900–3000 Da.

Peptide Mass Fingerprint identification of proteins was performed by searching against the *Glossina* entries of either the Swiss-Prot or TrEMBL databases (http://www.expasy.ch) and by using the MASCOT v2.3 algorithm (http://www.matrixscience.com) with trypsin enzyme specificity and one trypsin missed cleavage allowed [33]. Carbamidomethyl was set as fixed cystein modification and oxidation was set as variable methionine modification for searches. A mass tolerance of 50 ppm was allowed for identification. Matching peptides with one missed cleavage were considered as pertinent when there were two consecutive basic residues or when arginine and lysine residues were in an acidic context. MASCOT scores higher than 47 were considered as significant (*p <* 0.05) for Swiss-Prot and TrEMBL (v 2011_04) database interrogations.

#### Immunoblotting and specific proteins identification

The proteins separated by 2DE were then electro-transferred onto a PVDF (polyvinylidene dilfluoride, Biorad) membrane for western blotting as previously described [34]. The PVDF membrane was washed in Tris Buffer Saline (TBS) and then incubated in blocking buffer (TBS tween 0.05 % and 5 % dry milk) for 1 h 30 min. After washing three times with TBS tween 0.1 %, the membrane was incubated overnight at 4 °C with bovine serum diluted at 1/100 in TBS tween 0.05 % with 2.5 % dry milk. The membranes were then washed three times with TBS tween 0.1 % and three times with TBS, and then equilibrated with 2.5 % dry milk in TBS for 15 min. Mouse anti-bovine IgG conjugated to peroxidase (Sigma, St Louis, MO, USA) was added at a dilution of 1/15 000 in TBS tween 2.5 % with 2.5 % dry milk for 2 h 30 at room temperature. After addition of the secondary antibody, the membranes were washed four times with TBS tween 0.1 % and four times with TBS. The immunogenic proteins in membranes were revealed using the West Pico ECL (Pierce, Rockford, IL, USA) and exposed to XCLXposure films (Pierce) for 4 min.

Digital images of both western blotting and 2D gel were captured by scanning at 16 bits resolution under non saturating conditions, 300 dpi and stored in TIFF format. The proteins visualised on 2D gels were matched with immunogenic proteins detected by western blotting using SameSpots^TM^ Software 3.3 (Nonlinear Dynamics) in order to identify immunogenic spots.

### Peptide design

The sequences of proteins of interest were downloaded on the ExPASy Proteomics Tools server (http://www.expasy.ch). The signal peptides were predicted by SignalP 4.0 [35] and cleaved from the immature protein sequences. Secondary structures were then predicted with NetSurfP [36] and I-Tasser [37] servers. The surface exposure of proteins of interest has been viewed in 3D by the Pymol software (http://www.pymol.org).

The identification of putative linear B-cell epitopes of identified proteins was carried out with Bcepred [38], Bcpred [39] and Antigenicity plot server [40]. All epitopes that were identified by at least two out of three algorithms were selected. Peptides having a length less than 30 amino acids and including the maximum of epitopes were selected for further analyses. The specificity of peptide sequences to the *Glossina* genus was checked by requests on the NCBI Blast T non redundant databases (http://www.blast.ncbi.nlm.nih.gov/Blast.cgi). Synthetic peptides were synthesized and purified (95 %) by Genepep SA (St-Jean de Vedas, Montpellier, France). All peptides were shipped in lyophilised form and were then resuspended in ultrapure water (1 mg/ml) and stored as frozen aliquots.

### Evaluation of bovine IgG Ab level against synthetic peptides and WSE

The anti-peptide IgG responses were measured by indirect-ELISA, according to Poinsignon et al. [22] with minor modifications. Briefly, microtiter plates Maxisorp (Nunc, Roskilde, Denmark) were coated with peptide (20 μg/mL) in phosphate buffer saline (PBS) for 2 h 30 min at 37 °C. After three washes, plates were saturated with blocking buffer (Pierce, thermo scientific) 1 h at 37 °C. Sera diluted in PBS-tween 1 % (1/30 for Tsal1_52–75_ and 1/10 for Tsal1_145–166_) were incubated overnight at 4 °C. After five washes, sheep anti-bovine IgG conjugated to peroxidase (AbD Serotec, France) was added (in dilution 1/4000 for Tsal1_52–75_ and 1/2000 for Tsal1_145–166_) in PBS tween 1 % for 1 h 30 at room temperature. Colorimetric development was carried out using ABTS (2,2′-azino-bis (3-ethylbenzthiazoline 6-sulfonic acid) diammonium) (Sigma St Louis, MO, USA) in 50 mM citrate buffer (pH 4) containing 0.003 % H_2_O_2_. Optical density (OD) was measured at 405 nm (45 min for Tsal1_52–75_ and 1 h for Tsal1_145–166_). IgG responses against WSE were also evaluated on the same samples as described previously [19]. Each test sample was analysed in duplicate in antigen wells and, in parallel, in a blank well containing no peptide solution or no WSE (ODn) to control non-specific reactions between the serum and the reagents. Individual results were expressed as ∆OD value calculated according to the formula ∆OD = ODx - ODn, where ODx represents the mean of individual OD in both antigen wells, as previously used [22].

### Statistical analysis

All data were analysed with GraphPad Prism5 software® (San Diego, CA, USA). After verifying that ∆OD values did not assume Gaussian distribution using Shapiro-Wilk test, the non-parametric Mann–Whitney U-test was used for comparison of Ab levels between two independent groups. All differences were considered significant at *P  <* 0.05.

## Results

### Identification of specific immunogenic salivary proteins of *Glossina* exposure

**Fig. 1:**
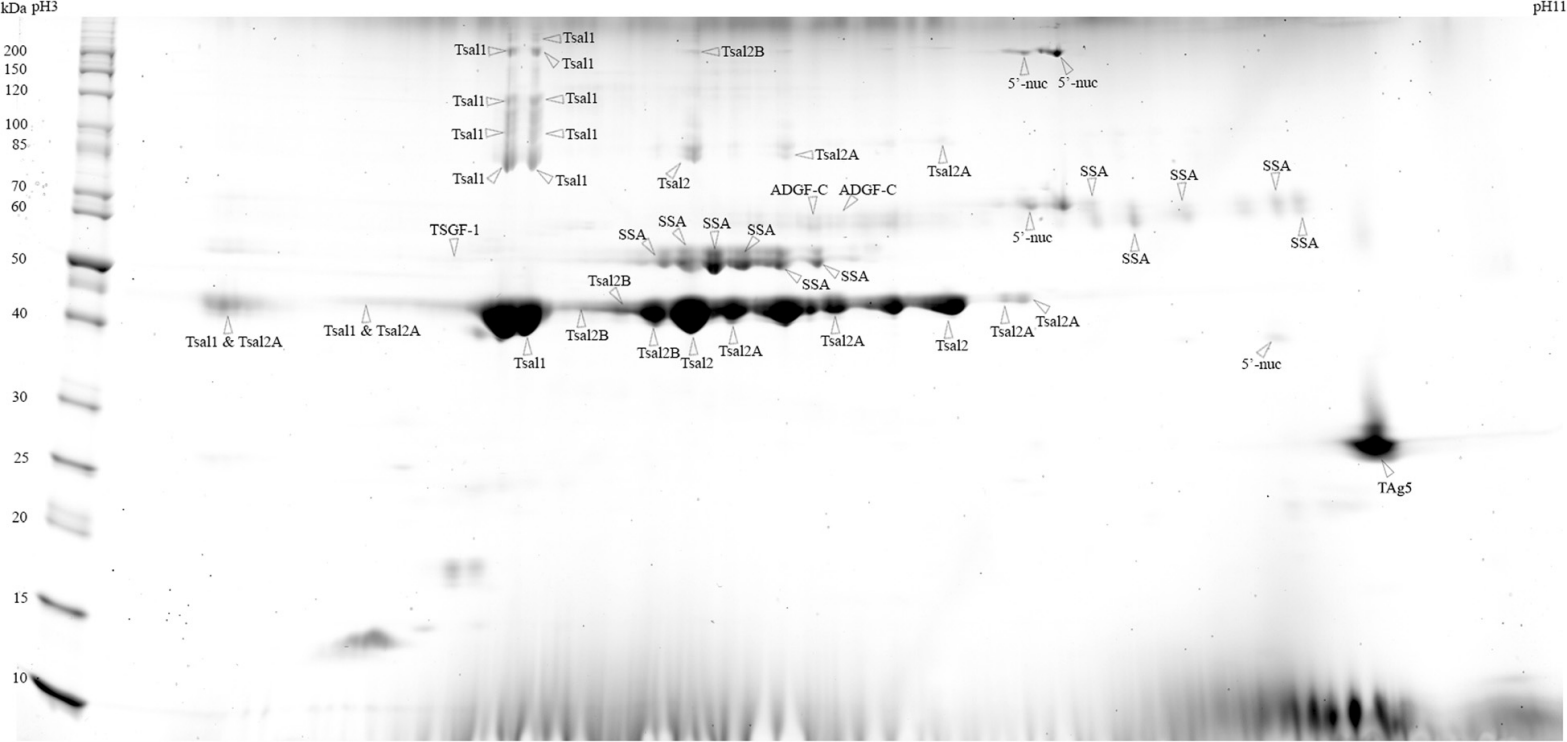
2D gel profile (SDS-PAGE) of *Glossina morsitans submorsitans* secreted salivary proteins. Whole saliva extracts were run on 2DE gels and stained with colloidal blue. Fifty-three spots were analysed by mass spectrometry and 47 lead to an identification. Molecular weight markers (MW) are indicated on the left. Abbreviations: Tsal1 (Tsetse salivary gland protein 1), Tsal2 (Tsetse salivary gland protein 2), Tsal2A (Tsetse salivary gland protein 2, isoform A), Tsal2B (Tsetse salivary gland protein 2, isoform B), TSGF-1 (Tsetse Salivary Growth Factor 1), SSA (Salivary Secreted Adenosine), ADGF-C (Adenosine deaminase-related growth factor C), 5′-nuc (5′nucleotidase family salivary protein) and TAg5 (Tsetse Antigen 5)

The sialome of *G. m. submorsitans* was investigated by 2D-gel electrophoresis separation of the WSE followed by colloidal blue staining (Fig. 1) and subsequent MALDI-TOF/MS identification. A total of 53 spots were observed with colloidal blue staining and could be picked for mass spectrometry analysis. Forty-seven spots were successfully identified by MALDI-TOF MS analysis providing a catalogue of seven salivary proteins (Table 1). For the spots of low molecular weight (< 25kD) no identification could be obtained because the amount of material was too scanty. In contrast, low molecular weight spots are much more intense in 2-D electrophoresis gels obtained with *G. m. morsitans* saliva extracts obtained by centrifugation of salivary glands [41]. These differences may be related to differences in the saliva composition of these two closely related subspecies but also to the saliva collection technique. Several spots with similar molecular weight but different isoelectric points led to the same identification suggesting the existence of isoforms for these proteins. All spots were identified as related to *G. m. morsitans* salivary proteins [41]: the Tsetse salivary gland protein 1 (Tsal1); the Tsetse salivary gland protein 2 (Tsal2) and its two isoforms (Tsal2A and Tsal2B); the Tsetse Salivary Growth Factor 1 (TSGF-1); the Salivary Secreted Adenosine (SSA); the Adenosine Deaminase-related Growth Factor C (ADGF-C); the 5′Nucleotidase family salivary protein (5′-nuc) and the Tsetse Antigen 5 (TAg5).

**Table 1:** *Glossina morsitans submorsitans* salivary secreted proteins identified by mass spectrometry. Database searches were performed against the *Glossina* entries of the SwissProt or TrEMBL databases with the MASCOT software. Molecular mass, pI and sequence coverage are shown. All the MASCOT scores are > 47 (*p <* 0.05)

Abbreviation	Protein identification	Protein family	Swissprot-TrEMBL accession number	Mass (kDa)		pI	Coverage (%)	Mascot score	Length (amino acids)	Functions
				Exp	Theo					
Tsal1	Tsetse salivary gland protein 1, *G. m. m.*	Endonuclease	D3TS87_GLOMM	46.155	45.613	5.06	13	79	399	Endonuclease activity and blood meal digestion
Tsal2	Tsetse salivary gland protein 2, *G. m. m.*	Endonuclease	D3TMW5_GLOMM	45.550	43.956	5.74	28	68	388	Endonuclease activity and blood meal digestion
Tsal2A	Tsetse salivary gland protein 2, isoform A, *G. m. m.*	Endonuclease	A3FMN3_GLOMM	44.601	44.002	6.16	22	76	388	Endonuclease activity and blood meal digestion
Tsal2B	Tsetse salivary gland protein 2, isoform B, *G. m. m.*	Endonuclease	A3FMN4_GLOMM	44.567	43.968	5.74	20	68	388	Endonuclease activity and blood meal digestion
TSGF-1	Tsetse Salivary Growth Factor-1, *G. m. m.*	Salivary adenosine deaminase (ADA)	D3TLK6_GLOMM	56.783	56.591	5.52	44	245	494	Vasolidation and platelet anti-aggregating
SSA	Salivary Secreted Adenosine, *G. m. m.*	Salivary adenosine deaminase (ADA)	D3TQW6_GLOMM	41.309	41.222	9.94	12	82	349	Vasolidation and platelet anti-aggregating
ADGF-C	Adenosine deaminase-related growth factor C, *G. m. m.*	Salivary adenosine deaminase (ADA)	DT3QW4_GLOMM	62.390	62.201	7	19	99	535	Vasolidation and platelet anti-aggregating
5′-nuc	5′nucleotidase family salivary protein, *G. m. m.*	5′nucleotidase/Apyrase	D3TRV7_GLOMM	62.479	62.062	7.19	16	75	555	ATP-diphosphohydrolase activity
TAg5	Tsetse Antigen 5, *G. m. m.*	Antigen 5 (AG5) family	Q9NBA6_GLOMM	29.647	28.925	8.58	40	113	295	Hypersensitivity I reaction and anti-hemostatic activity

*G. m. m.: Glossina morsitans morsitans,* Exp : experimental and Theo : Theoretical

**Fig. 2:**
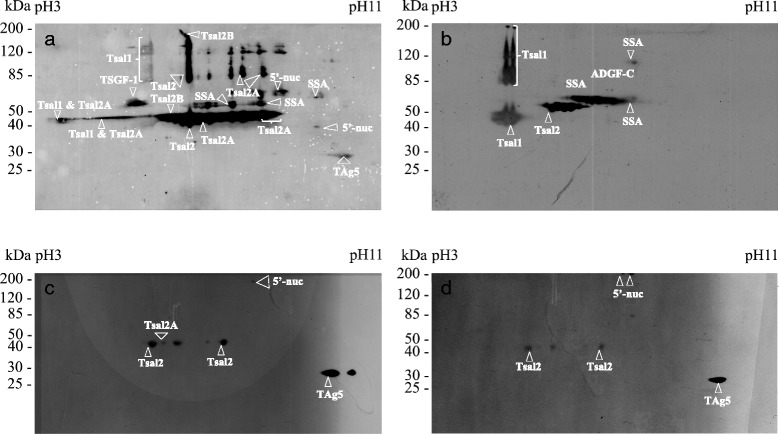
*Glossina morsitans submorsitans* immunogenic salivary proteins in cattle. *G. m. submorsitans* whole salivary extracts were run on 2D gels and transferred to PVDF membranes. Membranes were then incubated with sera from cows experimentally bitten by (**a**) *G. m. submorsitans*, (**b**) *G. p. gambiensis*, (**c**) *An. gambiae* and (**d**) *Stomoxys spp*. Molecular weight markers (MW) are indicated on the left

In order to identify *G. m. submorsitans* immunogenic salivary antigens that are specific of cattle exposure to tsetse flies, we established the 2D immunoblot profiles of serum samples from cows experimentally exposed to tsetse flies (*G. m. submorsitans*, *G. p. gambiensis*) or to other bloodsucking arthropods that are common in the study area (*A. variegatum*, *An. gambiae*, *Tabanidae* spp. or *Stomoxys* spp.). A number of spots were common between the two tsetse species but differences could be observed (Fig. 2). Whereas ADFG-C appears to be immunogenic in the cow exposed to *G. p. gambiensis*, this salivary protein did not react with the *G. m. submorsitans* serum. On the contrary, the *G. p. gambiensis* serum did not recognize any spot associated with the Tsal2 isoforms (Tsal2A and Tsal2B), TSGF-1, 5′-nuc and TAg5 families of salivary proteins. Importantly, cross reactions (although weak) were also observed with the sera of cows experimentally exposed to *Stomoxys* spp. or *An. gambiae* which recognised Tsal2, 5′-nuc, TAg5 and Tsal2, Tsal2A, 5′-nuc, TAg5 respectively (Table 2). Based on these results we decided to focus the peptide design on Tsal1 and SSA sequences as these salivary antigens were recognised only by the sera of animals individually exposed to both tsetse species and did not react with the sera of animals bitten by the other arthropod species.

**Table 2:** *Glossina morsitans submorsitans* salivary secreted proteins recognised by cows exposed to tsetse and other hematophagous arthropods

Arthropod species used for experimental exposure	Tsal1	Tsal2	Tsal2A	Tsal2B	TSGF-1	SSA	ADGF-C	5′-nuc	TAg5
*G. m. submorsitans*	+	+	+	+	+	+	-	+	+
*G. p. gambiensis*	+	+	-	-	-	+	+	-	-
*Stomoxys* spp.	-	+	-	-	-	-	-	+	+
*An. gambiae*	-	+	+	-	-	-	-	+	+
*Tabanidae* spp.	-	-	-	-	-	-	-	-	-
*A. variegatum*	-	-	-	-	-	-	-	-	-

‘+’ indicates *Glossina morsitans submorsitans* salivary proteins recognised on 2D gels by western blot with sera from animals exposed experimentally to several arthropod species

### Peptide design

**Fig. 3:**
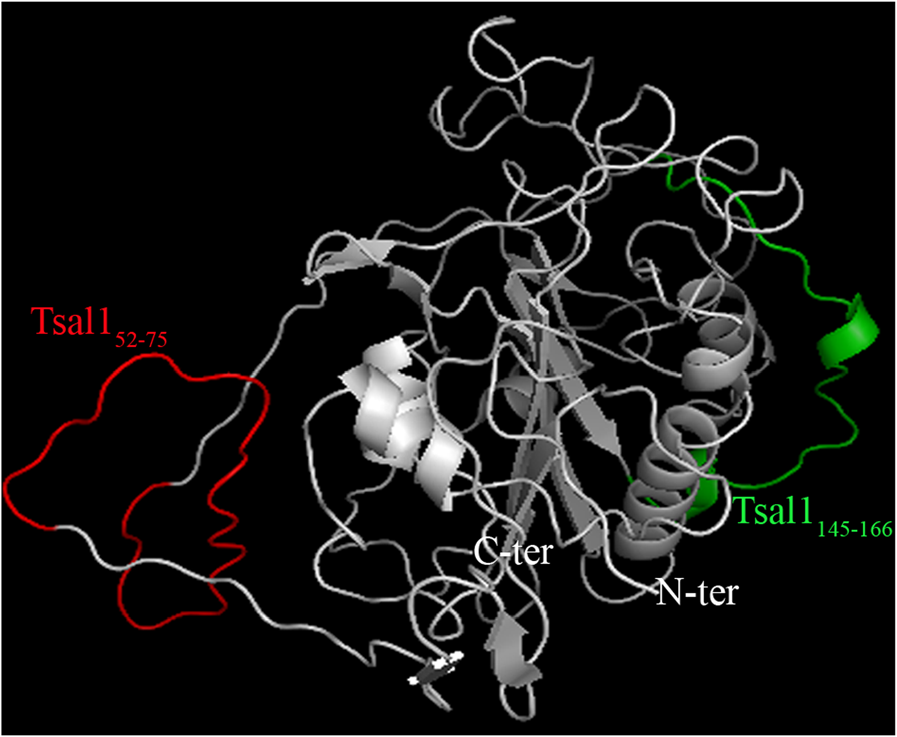
Tsal1 3D prediction model. The image was generated by the Pymol software (http://www.pymol.org) from the most probable structures published on the I-Tasser server [37]. N-ter is the first amino acid of the protein and C-ter, the last. Candidate biomarker peptides are colored in red for Tsal1_52–75_ and green for Tsal1_145–166_

Four and one sequences of 22 to 28 amino acid residues containing putative linear epitopes were identified from the *G. m. morsitans* Tsal1 and SSA sequences respectively (Additional file 1: Table S1). In order to avoid potential cross-reactivity with Abs against proteins from other bloodsucking arthropod species as well as from host pathogens, these 5 peptides were submitted to the NCBI Blast T non redundant databases (Additional file 1: Table S1). The SSA_65–92_ peptide showed some degree of homology with *Culex quinquefasciatus* and was therefore dropped out in further analyses. Similarly, among the 4 peptides identified to carry putative Tsal1 linear epitopes, we selected Tsal1_52–65_ and Tsal1_145–166_ as for the other two peptides, closest matches were obtained for organisms to which cattle may be naturally exposed (*Plasmodium yoelii yoelii* and *An. darlingi*). After checking for Ab accessibility of these two peptides at the surface of the Tsal1 protein using 3D models (Fig. 3), Tsal1_52–75_ and Tsal1_145–166_ were synthesised and tested against cattle serum samples.

### Ability of synthetic peptides to detect exposure to tsetse flies

**Fig. 4:**
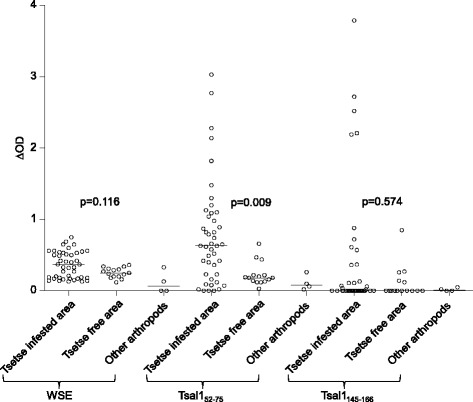
Cattle IgG responses against WSE and, Tsal1_52–75_ and Tsal1_145–166_ peptides_._ The IgG responses directed against whole saliva extracts (WSE) and the two candidate synthetic peptides were investigated in 43 animals from a tsetse infested area, 17 animals from a tsetse free area and four animals exposed experimentally to *A. variegatum*, *An. gambiae*, *Tabanidae* spp. or *Stomoxys* spp. (other arthropods). Individual ∆OD values are represented by empty circles. In the scatter plot, the horizontal bars indicate the median value for each group. Statistical significance between the different groups is indicated (non-parametric Mann–Whitney U-test)

The ability of the two synthetic peptide candidates to detect exposure of cattle to tsetse flies was first assessed on serum samples from cattle bred in different eco-climatic zones in Burkina Faso. Serum samples from the cows experimentally exposed to the none-tsetse species were also included as negative controls (Fig. 4). Anti-Tsal1_52–75_ IgG responses were significantly higher (*P  =* 0.009) in cattle from tsetse infested area as compared to those from tsetse free area, and were the lowest in the control animals exposed to tabanids, stable flies, mosquitoes or ticks. They also provided a better discrimination between animals from tsetse infested or free areas as compared to the response directed against WSE (*P  =* 0.116). In contrast no significant differences in the IgG response specific to Tsal1_145–166_ (*P  =* 0.574) were observed according to the exposure status, and this peptide was dropped out in further analyses. This may be due to the fact that as shown in Fig. 3 the Tsal1_145–166_ sequence is predicted to be involved in the formation of Tsal1 secondary structures, which may have altered its immunogenic properties.

**Fig. 5:**
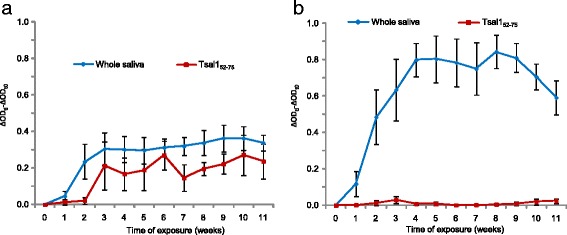
Monitoring anti-Tsal1_52–75_ and anti-tsetse saliva antibody responses in cows experimentally exposed to low and high levels of tsetse bites. **a** Low exposure group (10 flies weekly) and (**b**) high exposure group (50 flies twice a week). Vertical bars above or below the curves are the standard errors of the group mean

Finally the performance of the Tsal1_52–75_ peptide as a biomarker of exposure was further explored with experimental sera obtained from cows exposed to low or high regimen of tsetse bites (Fig. 5). In the low exposure group (10 flies/once a week), an early increase of the specific IgG response was observed after only three weeks of exposure and was maintained over the exposure period (11 weeks). Unexpectedly, very low IgG Ab levels were observed in the high exposure group (50 flies/twice a week), throughout the exposure period despite the fact that these animals mounted high IgG responses to WSE.

## Discussion

In this study, immuno-proteomics and bioinformatics tools were combined to design specific peptides as potential biomarker candidates for evaluating cattle exposure to tsetse flies. Indirect-ELISA tests using the identified peptides as antigens were then performed on sera from cattle exposed naturally or experimentally to tsetse bites. The Tsal1_52–75_ peptide appears as a promising candidate as anti-Tsal1_52–75_ IgG responses were detected in both naturally exposed animals and in cows submitted to low tsetse exposure levels that where close to the tsetse challenge observed in the study area [42].

A number of tests have been developed to assess host exposure to tsetse bites. Early studies focused on the detection of host Abs raised against WSE from several tsetse species: *G. p. gambiensis* to assess human exposure in West Africa [18]; *G. m. submorsitans* to assess cattle exposure in West Africa [19]; *G. f. fuscipes* to assess human exposure in Central Africa [16, 17]; and *G. m. morsitans* to assess human exposure in East Africa [15]. These studies showed that proteins from the Tsal family are major constituents of tsetse saliva, and induce strong Ab responses in tsetse exposed hosts. These proteins were thus considered as interesting candidates to develop biomarkers of tsetse exposure. Consecutively, it was shown that a *G. m. morsitans* Tsal1 recombinant proteins could be used instead of WSE in mice and pigs experimentally exposed to tsetse flies [15, 26]. Because the production of Tsal recombinant proteins in large quantities is difficult, possibly due to the DNA binding/endonuclease activity of Tsal proteins [43], these authors also developed a nanobody-based competitive immunoassay to detect anti-Tsal Abs [44]. The advantage of this method is that the same test can be applied to a wide range of hosts; nevertheless the test still requires the use of WSE, the production/storage of which can be a limitation in the context of laboratories from developing countries. In the present study, we evaluated the immunogenic properties of *G. m. submorsitans* salivary antigens in cows exposed to two tsetse species and to other bloodsucking arthropods. Whereas *G. m. submorsitans* Tsal proteins were shown to be highly immunogenic, only anti-Tsal1 IgG Abs were specific to tsetse fly exposure as immune cross-reactions with Tsal2 proteins were observed in animals exposed to stable flies or *An. gambiae*. The results presented in the present work point out Tsal1 as the best salivary antigen candidate to develop a highly specific biomarker of cattle exposure. Nevertheless, a study carried out on humans led to different results [18] as both Tsal proteins were recognised by sera from unexposed individuals. Instead this work led to the identification of a specific epitope within the TSGF-1 salivary protein [29], which has now been validated to monitor human exposure to *G. p. gambiensis* during a vector control campaign in Guinea [30]. It is noteworthy that in the present study, Ab response was detected against TSGF-1 only in the cow exposed to *G. m. submorsitans*. This illustrates that results obtained in a given animal model cannot always be extrapolated to another. These differences are likely due (i) to the sequence diversity of salivary proteins between the different tsetse species; (ii) to host species specificities in immune recognition; but also (iii) to the range/level of biting insects or pathogens to which tsetse hosts are submitted to and that can vary greatly between mammals or eco-climatic contexts. Hence, available serological tools, especially those relying on the recognition of a limited number of epitopes (such as it is the case for recombinant proteins and synthetic peptides), should be carefully evaluated prior to implementation as specificity and sensitivity of a given test may vary greatly according to the different contexts.

Tsal1_52–75_-based immunoassays, appear as promising tools to assess cattle exposure in West Africa where *G. morsitans* and *G. palpalis* subspecies represent the main tsetse species. Because this peptide was designed from the *G. m. morsitans* Tsal1 sequence, it might also be applied more widely as suggested by the results obtained experimentally on mice and pigs with the *G. m. morsitans* r-Tsal1 protein [26]. A surprising result was the fact that in our experimental conditions, no Ab response to Tsal1_52–75_ was observed in the group of cows submitted to intensive tsetse fly bites despite the fact these cows exhibited strong Ab responses against WSE. This suggests that single epitopes behave differently in terms of immunogenicity according to the exposure conditions. Noteworthy, r-Tsal1 indirect-ELISA tests [26] or Tsal specific monoclonal nanobodies [44] were also less efficient than WSE to discriminate between mice or pigs exposed to different biting regimens. According to our results, the anti-Tsal1_52–75_ Ab response represents a biomarker of low exposure levels but is likely less useful to measure the intensity of cattle exposure. The mechanisms underlying this intriguing result are not yet understood but could be related to antigen specific B cell exhaustion or anergy induced by high antigenic stimulation levels. This is however, an interesting feature for a biomarker candidate as it suggests that the development of a qualitative Tsal1_52–75_ synthetic peptide-based immunochromatographic rapid test to detect low tsetse exposure levels is a reachable goal.

Declaring tsetse free areas or detecting the possible reemergence or reintroduction of tsetse flies after interventions is an important aspect of tsetse eradication campaigns [45]. Using tsetse traps only is challenging because this entomological method is not sensitive, even less when tsetse densities are low [8]. Hence in such context, it underestimates the true tsetse density or incorrectly concludes to the absence of flies. Serological tests able to detect low exposure levels could thus represent important alternative and complementary tools. Such tests could be used in the field on cattle herds or sentinel animals that are mobile baits naturally attractive for tsetse flies. Such sentinel animals are already commonly used in the frame of tsetse vector control campaigns to monitor trypanosome infections, an indirect marker of tsetse exposure. In our experimental conditions, the bite by less than 30 flies over a period of three weeks was sufficient to induce anti-Tsal1_52–75_ Ab responses. Further studies evaluating different biting regimens (number of flies, biting frequencies) as well as re-challenge experiments in previously exposed animals are required to determine more precisely the sensitivity of Tsal1_52–75_-based immunoassays. Indeed anti-saliva Abs were shown to be boosted by very low numbers of tsetse bites in re-challenged mice and pigs [26]. It will also be useful to determinate the persistency of Tsal1_52–75_ Ab after an exposure to tsetse bites.

## Conclusions

In conclusion, we identified a Tsal1 peptide whose Ab response is specific of cattle tsetse fly exposure. The IgG response directed to the Tsal1_52–75_ synthetic peptide could be a biomarker of low cattle exposure. These are promising results in the framework of developing simple Tsal1_52–75_ based immunoassays (such as rapid tests) to monitor the tsetse flies presence at low fly densities or to detect early reemergence in previously cleared areas.

## Additional file

Additional file 1: Table S1. Synthetic peptides and putative linear epitopes from *Glossina morsitans morsitans* Tsal1 and SSA salivary proteins. (DOCX 31 kb)
