# Supplementary material for: Identification of a Tsal152–75 salivary synthetic peptide to monitor cattle exposure to tsetse flies
Source: Parasit Vectors. 2016 Mar 15;9:149. doi: 10.1186/s13071-016-1414-8 (PMC4791801; doi:10.1186/s13071-016-1414-8)
Supplement: Additional file 1: Table S1. — Synthetic peptides and putative linear epitopes from Glossina morsitans morsitans Tsal1 and SSA salivary proteins. (DOCX 31 kb) [file 13071_2016_1414_MOESM1_ESM.docx]

**Supplementary table 1** Synthetic peptides and putative linear epitopes from *Glossina morsitans morsitans* Tsal1 and SSA salivary proteins.

| **Consensus peptides** | **E-value *G. m. morsitans*** | **Best match specie (E-value)** |
| --- | --- | --- |
| **Tsal1** |  |  |
| Sequence 1 _8-32_: 25 amino acids  **ESVENEK**TPVIMV**RK**SLKTFEYDLF | 10^-15^ | *Plasmodium yoelii yoelii* (4,5) |
| Sequence 2 _52-75_: 24 amino acids  TGAQNYF**KNGEESV**TLMC**HNNEFD✡** | **4.10^-15^** | **Human immunodeficiency virus 1 (15)** |
| Sequence 3 _83-107_: 25 amino acids DLFTCVK**TPTAELRKTKE**RCSLGDL | 7.10^-15^ | *Anopheles darlingi* (20) |
| Sequence 4 _145-166_: 22 amino acids  NGAAVN**YRVPESE**TDLTVSLAK**✡** | **4.10^-11^** | ***Harpegnathos saltator* (4,6)** |
| **SSA** |  |  |
| Sequence _65-92_: 28 amino acids  HFFKGKS**QMEND**SRVFRLLKNMPKGAAL | 2.10^-18^ | *Culex quinquefasciatus* (0,030) |

All peptides that were identified by at least two out of three epitope prediction algorithms (Bcepred, Bcpred and antigenicity plot) are listed for each protein. Amino acids in bold are those included in the linear epitopes. Each peptide was submitted to the NCBI Blast T non redundant database. Blast E-value for *Glossina morsitans morsitans* and the best match species are indicated. The **✡** sign indicates the candidate peptide selected for further biological validation.
